# Non-Tuberculous Mycobacteria at the Human–Animal–Environment Interface: Antimicrobial Resistance, Environmental Persistence and Cross-Species Exposure Risks

**DOI:** 10.3390/antibiotics15050467

**Published:** 2026-05-05

**Authors:** Irena Reil, Silvio Špičić, Sanja Duvnjak, Maja Zdelar-Tuk, Šimun Naletilić, Gordan Kompes, Maja Dopuđ

**Affiliations:** Department for Bacteriology and Parasitology, Croatian Veterinary Institute, 10000 Zagreb, Croatia; reil@veinst.hr (I.R.); spicic@veinst.hr (S.Š.); zdelar-tuk@veinst.hr (M.Z.-T.); kompes@veinst.hr (G.K.);

**Keywords:** non-tuberculous mycobacteria, One Health, antimicrobial resistance, environmental reservoirs, cross-species exposure, wildlife, zoonotic risk, public health

## Abstract

Background/Objectives: Non-tuberculous mycobacteria (NTM) are increasingly recognized as important opportunistic pathogens at the human–animal–environment interface. Their growing relevance is driven by increasing disease burden, environmental persistence, occurrence in multiple animal hosts and complex antimicrobial resistance (AMR) patterns. Unlike classical zoonotic pathogens, most NTM are primarily acquired from shared environmental reservoirs rather than through sustained host-to-host transmission. This review examines NTM from a One Health perspective, focusing on AMR, ecology, animal occurrence, cross-species exposure and public health relevance. Methods: A narrative review of the current literature was conducted to synthesize evidence on the ecology, environmental reservoirs, occurrence in animals, transmission patterns and AMR mechanisms of NTM. Particular attention was given to studies addressing the human–animal–environment interface and the implications of NTM for One Health surveillance and risk assessment. Results: The reviewed literature shows that NTM are widely distributed in water, soil, sediments and biofilms, creating repeated opportunities for exposure in both animals and humans. They have been reported in livestock, wildlife, companion animals, reptiles and aquatic organisms, where they may act as colonizers, opportunistic pathogens, or sources of diagnostic interference. Evidence for direct animal-to-human transmission remains limited, but animal and environmental findings are important for understanding ecological overlap, host range and circulation of resistant strains. AMR in NTM is shaped by intrinsic resistance, acquired mutations, efflux activity, and biofilm-associated tolerance, which together complicate treatment and resistance prediction. Conclusions: NTM should be considered environmentally maintained, multi-host organisms of increasing One Health importance rather than conventional zoonotic pathogens. Improved interdisciplinary surveillance, diagnostics and research are needed to clarify exposure pathways, resistance development and public health risk.

## 1. Introduction

Non-tuberculous mycobacteria (NTM) comprise a diverse group of mycobacterial species other than members of the *Mycobacterium tuberculosis* complex and *M. leprae* [1,2]. They are also commonly referred to as environmental mycobacteria because of their broad distribution in water, soil, sediments, dust and biofilms, and historically they were often described as atypical mycobacteria [1,2]. In contrast to tuberculosis and leprosy, which are caused by more host-adapted mycobacterial pathogens with distinct epidemiological, clinical and transmission patterns, NTM are primarily associated with environmental persistence and opportunistic infection. NTM disease therefore usually reflects environmental acquisition, marked species-level heterogeneity, and the frequent diagnostic challenge of distinguishing colonization or transient isolation from clinically significant infection [2,3,4].

NTM are increasingly recognized as opportunistic pathogens capable of causing a broad spectrum of disease in both humans and animals [2,5]. In humans, they most commonly cause pulmonary disease, but may also be associated with lymphadenitis, skin and soft tissue infections, musculoskeletal disease, and disseminated infections [3,4]. Disease occurs most often in susceptible hosts, particularly in older adults, patients with structural lung abnormalities, and individuals with impaired immunity [3,4]. Over the past two decades, the epidemiological importance of NTM has increased substantially in many regions of the world [6,7]. In several high-income countries, the incidence and prevalence of NTM disease, especially pulmonary disease, have shown a steady rise, while tuberculosis rates have declined or stabilized [3,6]. This trend has made NTM an increasingly important public health concern [3,4].

In animals, NTM may occur as environmental colonizers, opportunistic pathogens, or causes of granulomatous lesions, and they are also relevant because of their potential to interfere with bovine tuberculosis diagnostics [2,5]. A practical and still widely used distinction within the NTM group is the division into slow-growing and rapid-growing species according to time to visible growth in culture, although molecular and genomic methods are now central for accurate identification [4,8]. Rapid-growing species generally produce mature colonies within 7 days and include clinically important species such as *M. abscessus* and the *M. fortuitum* group, whereas slow-growing species include *Mycobacterium avium* complex (MAC), *M. kansasii*, *M. marinum*, and many others of clinical relevance [4,5,9,10]. This distinction remains useful because growth rate is linked not only to laboratory workflow but also to ecological behavior, clinical presentation, and broad therapeutic expectations [4,8].

The ecological success of NTM is closely linked to their remarkable ability to persist in natural and engineered environments, particularly in water distribution systems, household plumbing, hospital water networks, biofilms, soils, and aerosols [1]. These traits are supported by hydrophobic, lipid-rich cell envelopes, tolerance to disinfectants, adherence to surfaces, and biofilm formation, all of which facilitate survival under adverse environmental conditions and complicate control measures [1]. The role of animals in NTM epidemiology nevertheless remains complex and insufficiently resolved [2]. Numerous NTM species have been detected in domestic and wild animals, with implications for animal health, wildlife conservation, food production, and diagnostic interference with bovine tuberculosis surveillance [2]. However, the currently available evidence more strongly supports shared environmental exposure than sustained direct animal-to-human transmission for most NTM species [2,11].

A clear distinction between several frequently used but non-equivalent terms is therefore important for interpreting NTM within a One Health framework. Cross-species exposure refers to situations in which humans and animals encounter the same NTM species or strains through shared environmental reservoirs, whereas cross-species transmission implies direct or epidemiologically supported transfer between host species. Zoonosis is used here in the classical sense of infection naturally transmitted between vertebrate animals and humans, a standard that most NTM do not consistently meet [2,11]. Similarly, a reservoir denotes an ecological source in which organisms persist and from which infection may arise, whereas a sentinel host indicates environmental circulation or risk without necessarily maintaining the organism.

Another major reason for the growing importance of NTM is the difficulty of treatment and the substantial contribution of intrinsic and acquired antimicrobial resistance (AMR) to poor clinical outcomes [4,12]. Current therapy for NTM disease is prolonged, species-dependent, often poorly tolerated, and frequently only modestly effective [4]. International guidelines emphasize that treatment decisions must be individualized and based on species identification, disease phenotype, and susceptibility testing where relevant [4]. Even under guideline-based therapy, treatment often requires multidrug regimens lasting many months, while relapse, reinfection, and treatment failure remain major challenges [12]. These difficulties are driven in part by the intrinsic low permeability of the mycobacterial cell envelope, biofilm formation, efflux systems, inducible resistance mechanisms, and target-site mutations, all of which reduce the effectiveness of available drugs [12]. In this context, intrinsic resistance refers to baseline species-related reduced susceptibility determined by inherent structural or physiological features, acquired resistance refers to additional resistance emerging through mutation or acquired determinants under antimicrobial pressure, and tolerance refers to non-heritable survival under antimicrobial exposure, often associated with slow growth, stress adaptation, or biofilm-associated persistence [12,13].

NTM also deserve greater attention within the broader One Health AMR agenda [12,13]. Their ubiquity in water and soil, persistence in biofilms, frequent exposure to antimicrobial residues and disinfectants, and broad host range suggest that NTM may act as environmental sentinels of selective pressure and as reservoirs of difficult-to-treat phenotypes [1,12]. At the same time, interpretation of resistance in NTM remains challenging because susceptibility patterns are strongly species-specific, genotype–phenotype correlations are incomplete for many taxa, and genomic tools and resistance databases remain much better developed for *M. tuberculosis* than for the highly diverse NTM group [13]. Thus, AMR in NTM is not only a therapeutic problem but also an ecological and surveillance challenge that spans human, veterinary, and environmental microbiology [12,13].

In this review, we examine NTM through the lens of antimicrobial resistance and cross-species exposure at the human–animal–environment interface [2,12]. We focus on environmental reservoirs, occurrence in animals, evidence and limitations regarding cross-species transmission, major resistance mechanisms, and the public health implications of the increasing global burden of NTM disease [2,4]. Overall, the review positions NTM as environmentally maintained, multi-host organisms of growing One Health relevance rather than conventional zoonotic pathogens [1,2,12].

## 2. Methodology

This narrative review aimed to synthesize current evidence on NTM at the human–animal–environment interface, with emphasis on environmental reservoirs and persistence, animal occurrence, cross-species exposure risks, and AMR mechanisms and implications. Relevant conceptual, epidemiological, veterinary, environmental, and clinical microbiology literature was identified through structured searches of PubMed, Scopus, and Web of Science, supplemented by targeted manual screening of reference lists and forward citation searching of key publications.

The main structured literature search covered publications from January 2000 to March 2026, with the final search update performed on 24 April 2026. Older publications published before 2000 were additionally included when they were considered foundational or directly relevant for historical, conceptual, methodological, or mechanistic context. The search strategy combined terms related to NTM, environmental reservoirs, animal hosts, One Health, and AMR, including variants of “nontuberculous mycobacteria”, “environmental reservoirs”, “drinking water”, “biofilm”, “soil”, “aerosol”, “animal”, “livestock”, “wildlife”, “companion animals”, “aquatic organisms”, “One Health”, “antimicrobial resistance”, “intrinsic resistance”, “efflux”, “macrolide resistance”, “whole-genome sequencing”, and “genotype–phenotype”. Additional targeted searches were performed for clinically and epidemiologically relevant taxa and settings, including MAC, *M. abscessus*, *M. kansasii*, *M. marinum*, bovine tuberculosis diagnostic interference, hospital water systems, and *M. chimaera* heater–cooler device outbreaks. Representative search strings used for the database searches are provided in Supplementary Materials Table S1.

The core synthesis was based primarily on peer-reviewed original research and review papers published in English. However, because of the interdisciplinary and applied scope of the topic, the final narrative synthesis also included selected clinical guidelines, laboratory standards, public health reports, and other authoritative documents directly relevant to diagnostics, susceptibility testing, surveillance, and One Health interpretation. Exceptionally, editorials, commentaries, or brief reports were retained only when they provided specific contextual value for animal, environmental, or healthcare-associated NTM epidemiology.

Records identified through database and manual searches were assessed for thematic relevance, methodological clarity, and direct contribution to at least one of the core review domains: (i) NTM ecology and environmental persistence in natural or engineered systems; (ii) NTM detection, disease, or diagnostic interference in animals; (iii) evidence informing interpretation of cross-species exposure or transmission at the human–animal–environment interface; and (iv) AMR mechanisms, susceptibility patterns, and limitations of genomic resistance prediction. Studies focused exclusively on the *M. tuberculosis* complex or *M. leprae* were excluded unless they provided directly relevant comparative context for NTM epidemiology, diagnostics, vaccination interference, or resistance interpretation. Because this was a narrative review intended as an interpretative synthesis rather than a formal systematic review, PRISMA-based flow diagrams and quantitative study selection procedures were not applied, although the review process was guided by principles of transparency and methodological clarity appropriate for narrative reviews [14,15].

## 3. Ecology and Environmental Reservoirs of NTM

A defining feature of NTM is their strong ecological association with both natural and engineered environments [1]. Unlike obligate human pathogens, NTM are widely distributed in soils, sediments, surface waters, groundwater, drinking water systems, dust, and bioaerosols, where they persist as environmental colonists rather than incidental contaminants [1]. Their ecological success is largely explained by a lipid-rich, highly hydrophobic outer membrane that promotes surface attachment, resistance to desiccation, and tolerance to many chemical stressors [1]. This physiology, together with slow growth, oligotrophic adaptation, and biofilm formation, allows NTM to persist under fluctuating environmental conditions and to occupy niches that are difficult to eliminate by standard sanitation practices [1].

Water is considered the most important environmental reservoir of many clinically relevant NTM species [16,17]. Numerous studies have documented NTM in natural waters as well as in engineered water systems, including drinking water treatment plants, municipal distribution networks, household plumbing, hospital water systems, and showerheads [16]. Drinking water and premise plumbing have repeatedly been identified as exposure-relevant reservoirs for clinically significant NTM, and their abundance may be influenced by source water characteristics, treatment processes, distribution networks, and building-level plumbing conditions [16,17]. Importantly, NTM are not merely transiently present in these systems but are well adapted to survive and grow within them [17]. Their tolerance to low nutrient concentrations, elevated temperatures, and common disinfectants such as chlorine and chloramine gives them a selective advantage in treated water environments, especially where competing microorganisms are suppressed [16,17]. As a result, treated water systems can paradoxically become favorable ecological niches for NTM persistence [17]. A particularly important example of exposure from engineered water-associated systems is provided by *M. chimaera* contamination of heater–cooler devices used during cardiac surgery. These outbreaks demonstrated how NTM can persist in water-containing medical devices, become aerosolized, and subsequently cause delayed healthcare-associated infections, often recognized only long after exposure [18,19,20,21]. This case further illustrates the importance of water reservoirs, bioaerosol generation, and system design in NTM epidemiology beyond community and domestic settings [18,19,20,21].

Biofilm formation is central to NTM ecology and helps explain their long-term persistence in plumbing and other built environments [1]. NTM readily adhere to pipe surfaces, taps, showerheads, and storage tanks, where they form or integrate into multispecies biofilms [1,22]. Within biofilms, mycobacteria gain additional protection against disinfectants, hydraulic flushing, and other environmental stressors, while also benefiting from stable microenvironments and access to organic material [1]. This biofilm-associated lifestyle is especially important in hospital and household water networks, where repeated aerosol generation can create chronic opportunities for exposure [22]. In this context, NTM persistence is driven not only by resistance to water treatment but also by the architectural and operational features of engineered systems that support surface colonization and biofilm maturation [1].

An additional ecological factor relevant to NTM persistence in aquatic environments is their interaction with free-living amoebae. Several studies have shown that mycobacteria can survive within amoebae or co-occur with them in water systems and biofilms, suggesting that amoebae may function as environmental hosts, protective niches or vectors that support persistence under adverse conditions [23,24,25,26]. This interaction is potentially important not only for environmental survival but also for virulence, because intracellular adaptation within amoebae may select for traits that facilitate survival within mammalian macrophages. In this sense, amoebae have been proposed as environmental training grounds for intracellular pathogenicity in mycobacteria, reinforcing the idea that environmental ecology and host adaptation are closely linked in NTM biology [24,26].

Aerosolization from water is widely regarded as one of the principal exposure pathways for human NTM infection, particularly for pulmonary disease [1]. Showering, aerosol-generating plumbing fixtures, humidifiers, and medical water systems may all contribute to inhalational exposure, especially in indoor settings where susceptible individuals experience frequent or repeated contact [1]. This route of exposure is consistent with the predominance of pulmonary NTM disease and with the repeated recovery of NTM from showerheads and domestic plumbing [16]. Several studies have directly demonstrated NTM in household water and shower aerosols and shown transfer of viable NTM from shower water to indoor air, supporting the plausibility of inhalational exposure in indoor environments [27,28]. From a One Health perspective, aerosolizable water systems represent a point of convergence where environmental reservoirs intersect with human and animal exposure, especially in farms, veterinary settings, aquaculture systems, and shared domestic environments [2]. This built-environment pathway may be particularly relevant for susceptible hosts with frequent exposure, including individuals with chronic lung disease and immunocompromised patients [27].

Soil and dust also serve as important reservoirs of NTM, although their epidemiological role is more difficult to quantify than that of water [1]. Many NTM species are normal inhabitants of soils, peat, mud, and sediments, where they can persist under variable moisture, nutrient, and temperature conditions [1,2]. Soil disturbance, agricultural activity, animal movement, and wind-driven dust generation may all facilitate contact between environmental NTM and exposed hosts [2]. These reservoirs are particularly relevant for wildlife, grazing animals, and outdoor occupations, and they may help explain the wide host range observed for some NTM species [2]. At the same time, current evidence suggests that environmental overlap, rather than efficient host-to-host transmission, remains the dominant framework for understanding most NTM infections across species [2].

Environmental persistence of NTM has important implications for AMR and control [1,3]. The same structural and physiological traits that enable survival in water, soil, and biofilms also contribute to reduced susceptibility to disinfectants and antimicrobials [1]. Their impermeable cell envelope, aggregation behavior, stress tolerance, and efflux-associated defenses can promote survival after sublethal exposure to antimicrobials, biocides, and environmental contaminants [3]. In addition, persistence in complex multispecies biofilms may increase opportunities for selection under chronic low-level exposure conditions, even when direct horizontal gene transfer appears less central than in other bacterial groups [3]. Thus, NTM ecology cannot be separated from resistance ecology, as environmental persistence and treatment recalcitrance are closely intertwined [1,3].

Taken together, the ecology of NTM highlights their importance as environmentally persistent organisms with broad exposure potential across human, animal, and environmental compartments [2]. For this reason, environmental surveillance, water system management, and improved characterization of reservoir-specific NTM communities should be viewed as core components of future NTM risk assessment and prevention strategies [2].

**Figure 1:**
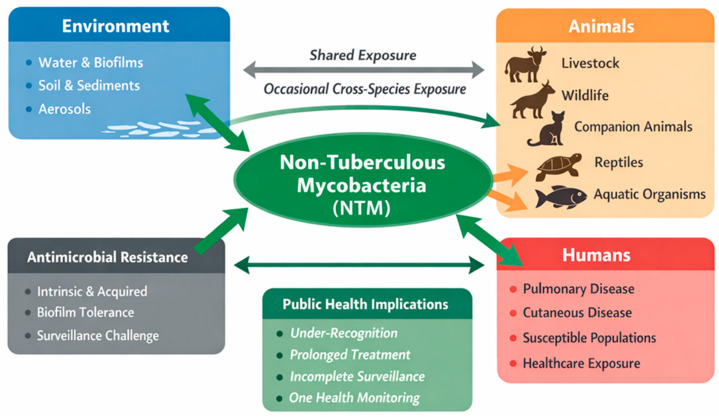
Conceptual One Health framework illustrating environmental reservoirs, animal hosts, human exposure, AMR, and public health implications of NTM.

A conceptual overview of environmental persistence, cross-species exposure, AMR, and public health relevance of NTM within a One Health framework is presented in Figure 1.

## 4. NTM in Animals

NTM have been reported in a wide variety of animal hosts, including livestock, companion animals, wildlife, birds, reptiles, and aquatic organisms, indicating a broad host range that reflects their strong environmental adaptability rather than strict host specificity [2,5]. In animals, NTM may be detected as environmental colonizers, opportunistic pathogens, or causative agents of clinically relevant granulomatous disease, depending on the host species, immune status, anatomical site, and infecting mycobacterial taxon [2,5]. At the same time, isolation of NTM from animal samples should be interpreted cautiously, since culture positivity does not always indicate active disease and may instead reflect exposure to shared environmental reservoirs [2,29].

Among domestic animals, NTM are particularly important because they can cause pathological lesions while also interfering with the diagnosis and surveillance of bovine tuberculosis [30]. Studies at the wildlife–livestock interface have shown substantial diversity of NTM species in cattle and wild animals, supporting the view that environmental mycobacteria may complicate tuberculosis control programs through cross-reactive immune responses and the presence of tuberculosis-like lesions [30]. Experimental work has further shown that some frequently recovered NTM species may contribute to false-positive or non-specific reactions in bovine tuberculosis testing, reinforcing the veterinary significance of these organisms beyond their direct pathogenicity [31].

Wildlife represents another important component of NTM ecology, because free-ranging animals are continuously exposed to soil, water, sediments, and other environmental sources where these organisms persist [2,29]. Recent studies from native wildlife have documented NTM in several wild species, indicating that contact with environmental mycobacteria is common even in conservation and pre-release settings [29]. Likewise, studies at the wildlife–livestock interface have demonstrated that multiple NTM species circulate among wild boar, cattle, and other hosts, which is more consistent with shared exposure within common habitats than with efficient host-restricted transmission [30]. This ecological overlap is especially relevant in One Health contexts where domestic animals, wildlife, and humans interact within the same landscapes and water systems [2,11].

Recent findings further illustrate the diversity and resistance profiles of animal-associated NTM [32,33]. Animal-derived NTM are also relevant from the perspective of AMR, as surveys of rapid-growing and slow-growing NTM isolated from domestic and wild animals demonstrated considerable resistance to multiple antimicrobial classes, supporting the view that animal-associated strains may represent reservoirs of difficult-to-treat phenotypes within the broader environmental resistome [32,33]. In addition, the isolation of multidrug-resistant *M. avium* subsp. *avium* from a wild Eurasian otter further expanded the spectrum of resistant mycobacteria reported from wildlife and underscored the importance of wild animals in the ecology of resistant NTM and related mycobacteria [34]. These findings are important within a One Health framework, although they should be interpreted primarily as evidence of ecological overlap, host breadth, and resistance circulation rather than as direct proof of zoonotic transmission [33,34].

Reptiles and aquatic organisms provide further examples of the broad animal distribution of NTM. NTM infections have been reported in captive and pet reptiles, indicating that these organisms can persist in exotic animal collections and private ownership settings where close human contact may occur [35]. In aquatic systems, mycobacteriosis is increasingly recognized as an important chronic disease of fish and other aquatic organisms, with several NTM species implicated in clinical disease, diagnostic challenges, and persistence in aquaculture and ornamental settings [36,37]. These hosts are particularly relevant because water-associated environments favor long-term persistence of mycobacteria and may facilitate repeated exposure of both animals and humans to the same strains or species [36,37].

Members of the MAC deserve particular attention in animal studies because they are among the most frequently detected NTM in both humans and animals and are widely distributed across environmental and host compartments [5]. MAC organisms have been described in birds, domestic animals, wildlife, and environmental matrices, which makes them especially relevant for discussions of cross-species exposure and ecological continuity between animal and human infection sources [5,38]. This is consistent with the broader concept that the animal significance of NTM lies not only in overt disease, but also in their ability to circulate across shared habitats and contribute to a common pool of environmentally maintained mycobacteria [2,38].

Overall, animal studies show that NTM have broad host range, substantial environmental connectivity, diagnostic significance, and relevant AMR potential [2,30,34]. For most species, however, these findings support shared environmental circulation more strongly than sustained animal-to-human transmission [11,29,33].

Selected NTM species of particular One Health relevance, together with their major reservoirs, animal hosts, public health significance, and resistance-related concerns, are summarized in Table 1.

## 5. Cross-Species Exposure and Transmission Potential of NTM

The question of cross-species transmission of NTM remains complex, and the available evidence does not support a simple classification of these organisms as classical zoonotic pathogens [2,49]. In contrast to members of the *M. tuberculosis* complex, most NTM infections are thought to arise from exposure to shared environmental reservoirs rather than from sustained transmission between hosts [1,50]. This distinction is central to the interpretation of NTM within a One Health framework, because the same species may be detected in humans, animals, water, soil, and biofilms without proving direct interspecies spread [2,5].

For most NTM species, the strongest epidemiological model is therefore one of shared environmental exposure rather than efficient host-to-host or animal-to-human transmission [1,11]. Animals, humans, and environmental compartments often overlap within farms, households, wildlife habitats, aquaculture systems, and water networks, creating repeated opportunities for exposure to the same mycobacterial species or strains [2,29]. Under such conditions, the recovery of related NTM from different hosts may reflect circulation within a common ecological niche rather than direct transmission from one host to another [30,38]. Animal-associated NTM should therefore be interpreted cautiously as indicators of ecological overlap and environmental circulation, rather than automatic evidence of reservoir status or zoonotic risk [2,33].

Nevertheless, some NTM species do have clearer zoonotic relevance than others. *M. marinum* is the best-recognized example, as it is a fish-associated NTM that can infect humans through skin trauma after contact with contaminated water, fish, or aquarium environments, producing the well-known fish tank granuloma [36,51]. In this case, the connection between aquatic animal infection, contaminated aquatic environments, and human disease is much more direct than for most other NTM species, although even here environmental mediation remains important [36,52]. Thus, *M. marinum* can be regarded as a genuine cross-species pathogen, but it is more the exception than the rule within the NTM group [36,51].

Members of the MAC are also highly relevant to discussions of cross-species transmission because they are widely distributed in humans, domestic animals, wildlife, birds, and environmental matrices [5,38]. However, the presence of MAC organisms across multiple host species does not by itself demonstrate direct interspecies transmission, since these bacteria are also abundant in water, soil, dust, and food-associated environments [5]. Current evidence therefore supports the interpretation of MAC as a group of environmentally maintained mycobacteria with broad host range and repeated opportunities for cross-species exposure, rather than as agents of frequent direct zoonotic spread in the classical sense [2,5].

Wildlife findings further reinforce this point. NTM have been detected in a variety of wild mammals and birds, but these observations are usually better explained by shared habitat exposure than by efficient wildlife-to-human transmission pathways [29,30]. Similarly, studies at the wildlife–livestock interface show that multiple NTM species may circulate among cattle, wild boar, and other free-ranging animals occupying the same environments, yet the available data rarely allow direct reconstruction of transmission chains between host species [30]. In practice, wildlife is more often part of a broader environmental network of exposure than a clearly demonstrated source of human infection [29,38]. Healthcare-associated exposure events further show that important NTM transmission pathways may arise from engineered environments rather than from classical zoonotic routes. The global *M. chimaera* heater–cooler device outbreaks associated with cardiac surgery demonstrated how contaminated water-containing devices can generate aerosols and expose patients to invasive NTM infection without direct animal involvement [18,19,20,21]. These events reinforce the broader concept that shared environmental and technical reservoirs are often more important than direct host-to-host transmission in NTM epidemiology [18,19,20,21].

From a public health perspective, this distinction is important. Overstating zoonotic transmission may misrepresent the biology of NTM and obscure the greater importance of water systems, biofilms, and environmental persistence in disease acquisition [1,2,11]. At the same time, underestimating cross-species relevance would also be inappropriate, because animal infections can signal the presence of contaminated environments, help identify shared ecological sources, and reveal antimicrobial-resistant strains of potential clinical relevance [32,34]. Animal findings are therefore valuable not necessarily because they prove direct zoonotic events, but because they strengthen surveillance at the human–animal–environment interface and improve understanding of how NTM circulate across ecological compartments [2,33].

An additional nuance is that transmission biology differs across the NTM group. While direct animal-to-human transmission remains poorly documented for most species, limited person-to-person transmission has been described for certain *M. abscessus* clones among patients with cystic fibrosis, showing that transmissibility cannot be generalized uniformly across all NTM [50,53]. Notably, genomic investigations in cystic fibrosis centers have provided evidence consistent with transmission of dominant *M. abscessus* clones between patients in some settings [54,55]. This does not make NTM broadly contagious, but it does underscore the need for species-level and context-specific interpretation when discussing transmission dynamics [49,50]. By analogy, the same caution should be applied to cross-species transmission claims involving animals, where broad ecological overlap may coexist with only rare or poorly documented direct transmission events [2,38].

Overall, current evidence supports the concept of cross-species exposure more strongly than epidemiologically important cross-species transmission for most NTM species, with selected exceptions such as *M. marinum* [2,11,36,51]. The main One Health challenge is therefore not to frame NTM as classical zoonoses, but to understand how environmental persistence, host breadth, and AMR create repeated opportunities for infection across shared ecological systems [1,13,38].

## 6. AMR

AMR is one of the central reasons why NTM have become an increasingly important clinical and One Health concern [12,13]. In contrast to many other bacterial pathogens, NTM are characterized by marked intrinsic resistance, highly variable species-specific susceptibility patterns, and a substantial capacity to acquire additional resistance during prolonged or repeated antimicrobial exposure [4,49]. As a result, treatment of NTM disease is often prolonged, toxic, expensive, and only moderately effective, with relapse, reinfection, and treatment failure remaining common outcomes in clinical practice [12,56].

A major component of resistance in NTM is intrinsic resistance, which is largely determined by the unusually hydrophobic, lipid-rich, and poorly permeable mycobacterial cell envelope [13]. This barrier limits antibiotic entry, reduces intracellular drug accumulation, and is further reinforced by a relatively low number of porin channels [13]. Efflux pumps also contribute to this baseline reduced susceptibility by actively exporting antimicrobial compounds from the bacterial cell, particularly in rapid-growing species such as *M. abscessus* [13,57]. Together, low permeability, limited uptake, and efflux activity create a background phenotype of reduced susceptibility that affects multiple antimicrobial classes and helps explain why NTM are inherently difficult to treat [12,13].

In addition to intrinsic resistance, NTM may develop acquired mutational resistance, particularly during prolonged or repeated therapy [12,13]. The best-characterized examples involve macrolides, aminoglycosides, and fluoroquinolones, although their importance varies across species [13,57]. In *M. abscessus*, inducible macrolide resistance is strongly associated with the presence and functionality of the *erm(41)* gene, whereas acquired high-level macrolide resistance is typically linked to mutations in the *rrl* gene encoding 23S rRNA [4,57]. Resistance to aminoglycosides such as amikacin may arise through mutations in the *rrs* gene, while fluoroquinolone resistance can involve mutations in *gyrA* and *gyrB*, although genotype–phenotype correlations are not equally robust across all NTM taxa [12,13]. These acquired mechanisms are clinically important because they can emerge under treatment pressure and may substantially reduce the effectiveness of key drugs within already limited therapeutic regimens [4,12,56].

A third important dimension is biofilm-associated tolerance, which is distinct from classical target-based resistance but highly relevant to treatment failure and environmental persistence [1,12]. NTM readily form or integrate into multispecies biofilms, where they gain additional protection against antibiotics, disinfectants, and other environmental stressors [1]. Within biofilms, mycobacteria benefit from protected microenvironments, altered growth states, and reduced antimicrobial penetration, all of which promote survival despite prolonged exposure [1,12]. This is particularly relevant for both clinical and environmental settings, as biofilm-associated persistence links resistance ecology with water systems, plumbing, healthcare-associated reservoirs, and other shared environmental compartments [1,3].

One of the major challenges in interpreting AMR in NTM is that susceptibility patterns are highly species-specific and cannot be generalized across the group [4,49]. Clinically important species such as MAC, *M. kansasii*, *M. abscessus*, and *M. fortuitum* differ substantially in intrinsic drug susceptibility, likelihood of acquired resistance, and expected treatment response [4,12]. This is why current international guidelines emphasize the need for accurate species-level identification and selective susceptibility testing rather than broad assumptions based on the label NTM alone [4,58]. Phenotypic susceptibility testing and interpretation are guided by standardized methods and recommendations [59,60,61], and ongoing EUCAST work is contributing to the development of (T)ECOFFs and clinical breakpoints for selected NTM–drug combinations [62].

Whole-genome sequencing (WGS)-based resistance prediction offers important opportunities for studying resistance in NTM, but its interpretation remains more difficult than in *M. tuberculosis* [12,13]. Resistance databases and genotype–phenotype frameworks are still far less developed for the highly diverse NTM group, and automated pipelines may miss clinically relevant determinants or fail to capture species-specific mechanisms [13]. Accordingly, discordance between phenotypic resistance and identifiable genomic determinants remains common in several NTM taxa, particularly among rapid-growing mycobacteria [63,64]. From a clinical perspective, this means that WGS-based resistance prediction in NTM cannot yet replace phenotypic susceptibility testing and should be interpreted together with species identification, phenotypic results, and current knowledge of resistance-associated loci [12,13,63,64]. This limitation is especially important for emerging animal and environmental isolates, where phenotypic resistance may be observed even when known genomic determinants are not readily identified [33,34].

Macrolides remain particularly important because they are cornerstone drugs in the treatment of many NTM infections, especially those caused by MAC and some rapid-growing mycobacteria [4,12]. Resistance to clarithromycin or azithromycin is therefore of major therapeutic significance and may have a stronger clinical impact than resistance to several other agents combined [4,56]. At the same time, interpretation of resistance should remain clinically grounded, since the significance of in vitro resistance differs according to species, drug class, inducible versus acquired mechanisms, and the structure of the treatment regimen as a whole [4,49].

The AMR problem in NTM also extends beyond human medicine. Their persistence in water systems, soil, sediments, biofilms, and animal hosts creates repeated opportunities for exposure to subinhibitory concentrations of antimicrobials, disinfectants, and other environmental stressors that may favor selection of tolerant or resistant phenotypes [1,12]. Animal-derived NTM isolates with resistance to multiple antimicrobial classes have already been documented, supporting the view that veterinary and wildlife settings may contribute to the broader ecology of resistance in these organisms [32,33]. Within a One Health perspective, these resistance mechanisms are particularly relevant because they operate across clinical, environmental, and animal-associated NTM populations exposed to shared selective pressures [1,32,33]. In this context, NTM may be viewed not only as opportunistic pathogens but also as environmental and animal-associated indicators of selective pressure operating across human, veterinary, and environmental compartments [13,49].

Overall, AMR in NTM is not a secondary feature but one of the defining characteristics of this group [12,13]. Intrinsic resistance, acquired mutational mechanisms, biofilm-associated tolerance, species-level heterogeneity, and incomplete genomic interpretability together make NTM exceptionally challenging organisms to treat and monitor [4,56]. Within a One Health framework, this means that AMR in NTM should be understood not only as a clinical treatment problem, but also as an ecological and surveillance issue that links environmental persistence, animal exposure, and public health risk across shared ecosystems [1,32,49].

Major resistance mechanisms in clinically relevant NTM species, together with the principal affected drug classes and therapeutic implications, are summarized in Table 2.

## 7. Public Health Implications

NTM are increasingly recognized as a growing public health concern because the burden of disease appears to be rising in multiple regions, particularly for pulmonary NTM disease in older adults and in patients with structural lung disease or immunocompromising conditions [6,7,49]. This trend is especially important because NTM disease is often chronic, difficult to diagnose, and associated with prolonged treatment courses and substantial healthcare utilization [3,56]. In practical terms, NTM therefore represent not only a microbiological challenge but also an increasing burden for respiratory medicine, infectious diseases, clinical microbiology, and public health surveillance systems [65,66].

One of the major public health difficulties is under-recognition. NTM disease is not uniformly reportable/notifiable in many jurisdictions, surveillance systems remain fragmented, and laboratory workflows are often not optimized for species-level monitoring across populations [49,65,66,67,68]. As a result, national estimates may be incomplete, temporal trends may be underestimated, and comparisons across regions are often complicated by differences in case definitions, diagnostic practices, and laboratory capacity [49,65]. The recent emphasis on strengthening NTM surveillance reflects increasing recognition that NTM should be addressed as a population-level health issue rather than only an individual clinical problem [65,66].

The diagnostic challenge is also considerable because isolation of an NTM from a specimen does not automatically indicate disease [4,56]. Accurate interpretation requires integration of microbiological findings with clinical symptoms, radiological evidence, and exclusion of alternative diagnoses, which creates a persistent risk of both overdiagnosis and underdiagnosis in routine care [4,56]. Delays in correct identification may affect individual outcomes and distort broader public health data on mycobacterial disease, particularly in settings where NTM may mimic tuberculosis clinically and microbiologically [3,69].

The treatment burden further increases the public health relevance of NTM. Current regimens are long, species-dependent, and often poorly tolerated, while treatment failure, relapse, and reinfection remain frequent even under guideline-based care [4,56,70]. Because successful management often requires prolonged multidrug therapy, radiological follow-up, and repeated culture monitoring, NTM disease places considerable demands on healthcare systems and multidisciplinary clinical teams [56,70]. AMR adds another major layer of public health importance, as NTM exhibit extensive intrinsic resistance, species-specific susceptibility patterns, and the capacity to acquire additional resistance during treatment [4,12,13,57].

From a public health perspective, the significance of NTM is also shaped by their environmental epidemiology. Because many infections are believed to arise from water, biofilms, soil, and aerosols rather than sustained person-to-person transmission, prevention strategies cannot rely only on classical infection-control logic used for contagious pathogens [1,14]. The global *M. chimaera* heater–cooler device outbreaks further highlighted the public health significance of NTM in engineered water systems, showing how environmental persistence, aerosolization, delayed diagnosis, and fragmented surveillance can converge in healthcare-associated exposure events [18,19,20,21]. This example illustrates the broader surveillance challenge posed by NTM, particularly when exposure arises from technical water-associated systems rather than from recognizable direct transmission chains [18,19,20,21]. Instead, public health approaches must also consider environmental monitoring, water system management, healthcare-associated exposure pathways, and risk communication for susceptible populations [1,56,66]. Animal and wildlife findings may further support risk assessment by identifying ecological hotspots, resistant phenotypes, and shared exposure settings at the human–animal–environment interface [2,34].

Overall, the public health importance of NTM lies in the convergence of rising disease burden, diagnostic difficulty, prolonged and resistance-limited treatment, incomplete surveillance, and persistent environmental exposure [56,65,66]. Their significance is therefore broader than the management of individual infections and increasingly relevant to surveillance policy, laboratory infrastructure, environmental health, and One Health risk assessment [3,49,65].

## 8. Knowledge Gaps and Future Directions

Despite growing interest in NTM, major knowledge gaps remain across epidemiology, diagnostics, AMR, and One Health surveillance [49,71]. One of the most important unresolved issues is that the true burden of NTM disease is still incompletely defined, largely because surveillance is inconsistent, case definitions are not always applied uniformly, and NTM infections are not systematically reported in many countries [49,65,67,68]. As a result, available incidence and prevalence estimates likely underestimate the actual burden and make comparisons between regions difficult [7,65].

A second major gap concerns the ecology of NTM at the human–animal–environment interface. Although environmental reservoirs such as water, soil, and biofilms are widely accepted as major sources of exposure, the relative contribution of specific reservoirs, exposure routes, and local ecological conditions remains poorly quantified for most NTM species [1,3]. The same applies to animal-associated NTM, where evidence strongly supports shared environmental exposure but is usually insufficient to distinguish between colonization, disease, and true transmission pathways [2,38]. Future studies should therefore move beyond simple detection and focus more on integrated ecological designs that combine environmental, veterinary, and clinical sampling within the same geographic systems [2,11].

Transmission is another area where terminology often exceeds the available evidence. For most NTM, direct animal-to-human transmission remains poorly demonstrated, and even when the same species is recovered from different hosts, this does not necessarily prove epidemiologically meaningful cross-species spread [2,30]. More comparative studies using genomics, source tracing, and shared-environment sampling are needed to distinguish true transmission events from parallel acquisition from common reservoirs [2,71]. In this respect, the field would benefit from greater use of the concept of cross-species exposure rather than assuming classical zoonotic transmission in the absence of stronger evidence [2,38].

Diagnostics remain another major bottleneck. Although molecular methods have greatly improved species identification, important challenges remain in discriminating closely related taxa, detecting mixed infections, standardizing workflows across laboratories, and linking identification results to clinical relevance [8,72]. Many diagnostic platforms are still optimized for *M. tuberculosis* rather than for the taxonomic breadth of NTM, and this affects both routine detection and resistance interpretation [8,73]. Future progress will depend on more accessible species- and subspecies-level diagnostics, better standardization of laboratory pipelines, and stronger integration of molecular results with clinical and ecological interpretation [8,72].

AMR in NTM also remains incompletely understood. While intrinsic resistance mechanisms such as low cell envelope permeability, efflux activity, and biofilm-associated tolerance are well recognized, genotype–phenotype correlations remain incomplete for many species and drugs [12,13]. Accordingly, discordance between phenotypic resistance and identifiable genomic determinants remains common in several NTM taxa, particularly among rapid-growing mycobacteria [63,64]. This is particularly problematic for whole-genome sequencing, where resistance databases and predictive frameworks are still much less developed for NTM than for *M. tuberculosis* [13,73]. As a result, phenotypic resistance may be observed without obvious genomic explanations, and clinically relevant determinants may be missed by automated pipelines designed around tuberculosis-focused databases [12,13]. Future work should therefore prioritize curated NTM-specific resistance databases, expanded genotype–phenotype studies, and validation of genomic markers across diverse species and ecological contexts [13,71].

Another important gap concerns standardization of antimicrobial susceptibility testing and interpretation. Although standardized methodological guidance is available [59,60,61], interpretive criteria remain incomplete for many NTM–drug combinations, limiting cross-study comparability [62]. Susceptibility patterns differ markedly across NTM species, and the clinical value of in vitro results varies according to the drug, species, and resistance mechanism involved [4,49]. This limits comparability between studies and complicates interpretation of AMR data from human, animal, and environmental isolates [12,33]. Greater harmonization of susceptibility testing methods, interpretive criteria, and reporting practices would strengthen both clinical management and surveillance-oriented research [4,13].

From a One Health perspective, one of the clearest future priorities is integrated surveillance. At present, human clinical data, veterinary findings, wildlife observations, and environmental monitoring are often generated in isolation, which makes it difficult to reconstruct how NTM circulate across shared systems [38,65]. Combining these data streams would improve identification of ecological hotspots, resistant phenotypes, and potentially shared sources of infection [2,34]. Such approaches are especially relevant in water-associated settings, wildlife–livestock interfaces, hospital water systems, aquaculture, and regions with rising pulmonary NTM disease burden [1,2]. Priority areas for future research include integrated One Health surveillance, harmonized susceptibility testing, NTM-specific resistance databases, and comparative genomic studies linking environmental, animal, and clinical isolates [2,13,34].

Another underappreciated One Health and public health issue is the possible influence of environmental NTM exposure on BCG vaccine efficacy. Exposure to NTM has long been proposed as one explanation for the variable protective effect of BCG observed across populations and geographic settings, because prior sensitization to environmental mycobacteria may alter vaccine take, block replication of the live vaccine, or skew subsequent immune responses [74,75,76]. Although the magnitude and mechanisms of this effect remain debated and appear to depend on the NTM species, route of exposure, and host context, the issue is highly relevant for tuberculosis control, vaccine evaluation, and interpretation of mycobacterial immune cross-reactivity in endemic settings [74,75]. Future One Health research should therefore consider whether environmental or animal-associated NTM exposure contributes to regional differences in BCG performance or interferes with the assessment of novel TB vaccines.

Finally, there is a need for greater conceptual clarity in how NTM are framed in the literature. They are often discussed either too narrowly as opportunistic clinical pathogens or too broadly as zoonotic threats, whereas the available evidence more strongly supports their interpretation as environmentally maintained, multi-host organisms with important AMR and public health implications [2,49]. A balanced research agenda should therefore avoid overstating direct zoonotic transmission while still recognizing that animal findings, environmental persistence, and resistant phenotypes are highly relevant to human health [32,34]. In future, the most productive advances will likely come from interdisciplinary studies that unite clinical microbiology, veterinary medicine, environmental science, genomics, and public health surveillance within a shared One Health framework [38,71].

## 9. Conclusions

NTM should be recognized as increasingly important opportunistic pathogens at the human–animal–environment interface [1,2]. Their significance lies in the convergence of environmental persistence, broad host exposure, complex and species-specific AMR, and a growing clinical burden that remains difficult to diagnose, treat, and monitor at the population level [12,49].

A One Health perspective is nevertheless highly appropriate for NTM because humans, domestic animals, wildlife, and aquatic organisms are all exposed to shared ecological reservoirs such as water systems, biofilms, soils, and sediments [1,2]. In this context, animal findings are especially informative not necessarily because they prove direct zoonotic transmission, but because they reveal environmental circulation, host breadth, and the presence of resistant phenotypes that may be relevant to public and veterinary health alike [32,34]. The concept of cross-species exposure therefore appears more accurate than generalized claims of cross-species transmission for most NTM species, with selected exceptions such as *M. marinum* illustrating that more direct zoonotic links can occur in particular ecological settings [2,36].

AMR remains one of the defining challenges in the NTM field because treatment outcomes are constrained by intrinsic resistance, acquired mutational mechanisms, biofilm-associated tolerance, and incomplete genotype–phenotype understanding across species [12,13]. These difficulties are compounded by fragmented surveillance and the fact that NTM are not routinely notifiable/reportable in many jurisdictions, although public health efforts to expand laboratory-based surveillance are increasing [65,66]. Future progress will depend on better species-level diagnostics, curated NTM-specific resistance databases, harmonized susceptibility testing, and integrated surveillance linking clinical, veterinary, and environmental data within shared epidemiological frameworks [4,13,66].

Overall, NTM should be viewed as a growing One Health concern whose importance extends beyond individual infections to broader questions of environmental persistence, AMR ecology, and public health preparedness [2,12]. A balanced interpretation avoids overstating classical zoonotic transmission while still recognizing that the interaction of environmental reservoirs, animal hosts, and human susceptibility creates repeated opportunities for infection across species boundaries [1,33]. In this sense, NTM offer a valuable model for understanding how environmental opportunists can become clinically and epidemiologically significant threats in an era of rising AMR and increasingly interconnected human, animal, and environmental health systems [49,66].

## Figures and Tables

**Table 1:** Selected NTM of One Health relevance: reservoirs, animal hosts, public health significance, and resistance-related concerns.

Species/Group	Main Environmental Reservoir(s)	Animal Hosts Reported	Human Relevance	Exposure/Transmission Note	AMR Relevance
MAC	Water, soil, dust, food-associated environments, biofilms	Birds, livestock, companion animals, wildlife	Major cause of pulmonary and disseminated NTM disease, especially in susceptible hosts	Broad multi-host distribution is well documented, but most evidence supports repeated environmental acquisition and cross-species exposure rather than frequent direct zoonotic transmission	Long multidrug regimens are often required; macrolides remain central, and resistance is clinically important when present [5,12]
*M. marinum*	Aquatic environments, aquaria, fish tanks, contaminated water	Fish and other aquatic organisms	Causes cutaneous infection in humans, classically “fish tank granuloma”	Best example of a more direct animal-/aquatic-associated cross-species pathogen among NTM, typically after skin trauma and contact with contaminated water or fish	Treatment is species-specific; clinically relevant because infection can be prolonged and require combination therapy [39,40,41]
*M. abscessus*	Water systems, biofilms, healthcare-associated water reservoirs	Occasionally detected in animals; most important as an environmental and clinical opportunist	Major rapid-growing NTM associated with severe pulmonary disease and difficult treatment	Usually interpreted as environmentally acquired, although limited person-to-person transmission has been described in selected clinical settings	One of the most drug-resistant NTM; resistance involves low permeability, efflux, inducible macrolide resistance, and biofilm-associated tolerance [9,12,42,43]
*M. kansasii*	Especially tap water and municipal water systems	Limited animal relevance compared with some other NTM; mainly discussed through environmental exposure	Important cause of pulmonary disease in humans	Environmental acquisition, particularly from water, is considered the dominant model rather than animal-mediated transmission	Clinically important because species-level identification influences regimen choice and interpretation of susceptibility data [10,44,45]
*M. fortuitum* group	Water, soil, dust, animal-associated environments, biofilms	Fish, domestic animals, wildlife, occasional broader veterinary reports	Opportunistic human pathogen, especially in skin/soft tissue and non-respiratory infections	Best interpreted as an environmental opportunist with broad ecological distribution rather than a classical zoonotic pathogen	Relevant because rapid-growing mycobacteria may show broad reduced susceptibility profiles and biofilm-associated persistence [46,47,48]
*M. malmoense*	Environmental reservoirs suspected, including water and soil, but ecology less clearly defined than for some other NTM	Wildlife and occasional animal reports, including recent Croatian findings	Recognized human pathogen, especially in pulmonary disease	Current evidence is more compatible with environmental exposure than proven animal-to-human transmission	Important mainly because resistant phenotypes may be detected despite incomplete genotype–phenotype understanding in NTM [2,12]
Animal-derived mixed rapid- and slow-growing NTM isolates (survey category) *	Water, soil, sediments, biofilms, shared farm/wildlife habitats	Domestic animals, wildlife, reptiles, aquatic organisms	Indirect public health relevance through ecological overlap, diagnostic interference, and resistant phenotypes	Animal findings usually support shared environmental circulation and cross-species exposure rather than direct proof of zoonotic transmission	Animal isolates can carry difficult-to-treat phenotypes and are useful for One Health AMR surveillance [32,33,34]

* This category refers to heterogeneous collections of environmental NTM reported from animals in surveillance or antimicrobial resistance studies rather than to a single defined species. In the present review, it mainly includes animal-derived rapid- and slow-growing isolates from recent surveys of domestic and wild animals, including mixed or unidentified NTM taxa in addition to species-specific findings discussed separately in the text [32,33,34].

**Table 2:** Major resistance mechanisms in clinically relevant NTM and their therapeutic implications.

Species/Group	Key Resistance Mechanism(s)	Major Drug Classes Affected	Clinical Implication
NTM (general)	Low cell envelope permeability, reduced drug uptake, limited porin-mediated entry, efflux pumps, biofilm-associated tolerance, target-site modification, occasional enzymatic inactivation	Multiple classes, depending on species	Intrinsic resistance is a defining feature of NTM and contributes to prolonged, difficult, and often only partially effective treatment regimens [1,12,13].
*M. abscessus*	Functional *erm(41)*-mediated inducible macrolide resistance; *rrl* mutations causing acquired high-level macrolide resistance; *rrs* mutations associated with aminoglycoside resistance; efflux activity; biofilm-associated tolerance; low permeability cell wall	Macrolides, aminoglycosides, β-lactams, fluoroquinolones, multiple additional agents	One of the most difficult NTM species to treat; species- and subspecies-level identification is essential, and macrolide susceptibility must be interpreted in light of inducible resistance testing or *erm(41)* sequencing [4,13,57].
MAC	Intrinsic low permeability, efflux-associated reduced susceptibility, biofilm-associated tolerance; acquired macrolide resistance may emerge under treatment pressure; species-specific variability in susceptibility	Macrolides, aminoglycosides, rifamycins, ethambutol and companion drugs within multidrug regimens	Macrolides remain cornerstone agents; macrolide resistance in MAC is clinically critical because it is strongly associated with poor outcomes and major therapeutic limitations [4,12,58].
*M. kansasii*	Species-specific susceptibility profile; clinically relevant resistance particularly interpreted for rifampicin; additional resistance patterns vary by isolate and setting	Rifampicin most clinically relevant, with additional implications for companion drugs	Accurate susceptibility interpretation is important because this species is often more predictably treatable than some other NTM, but rifampicin resistance has major therapeutic consequences [4,58].
*M. fortuitum* group	Intrinsic and acquired reduced susceptibility patterns; efflux, low permeability, and biofilm-associated persistence likely contribute; species/strain heterogeneity is substantial	β-lactams, macrolides, tetracyclines, fluoroquinolones and others depending on isolate	Rapid-growing mycobacteria such as *M. fortuitum* may show broad variability in susceptibility, so treatment selection should rely on isolate-specific testing rather than assumptions based on group-level behavior [12,13].
*M. marinum*	Species-specific susceptibility profile; resistance is generally less emphasized than ecological exposure and clinical recognition, but prolonged treatment may still be needed	Macrolides, rifamycins, ethambutol and companion agents depending on regimen	Public health importance lies more in recognizable aquatic exposure and delayed diagnosis than in extreme multidrug resistance, although correct species identification remains important for treatment planning [12,36].
Animal-associated rapid and slow-growing NTM isolates	Mixed intrinsic and acquired resistance patterns; phenotypic resistance may be present even when known genomic determinants are not easily identified; environmental selective pressures may contribute	Multiple classes, including cephalosporins, amoxicillin-clavulanate, doxycycline, rifampicin, ciprofloxacin, linezolid and others depending on species	Animal isolates are relevant for One Health surveillance because they may reveal circulation of difficult-to-treat phenotypes across veterinary, wildlife, and environmental compartments [32,33,34].

## Data Availability

No new data were created or analyzed in this study.

## Supplementary Materials

The following supporting information can be downloaded at: https://www.mdpi.com/article/10.3390/antibiotics15050467/s1, Table S1: Representative database search strings used in this narrative review.

## Abbreviations

The following abbreviations are used in this manuscript:

NTM	non-tuberculous mycobacteria
AMR	antimicrobial resistance
WGS	whole-genome sequencing
MAC	Mycobacterium avium complex
PRISMA	Preferred Reporting Items for Systematic Reviews and Meta-Analyses
EUCAST	European Committee on Antimicrobial Susceptibility Testing
(T)ECOFFs	tentative epidemiological cut-off values/epidemiological cut-off values

## References

[1] Falkinham J.O. (2021). Ecology of Nontuberculous Mycobacteria. Microorganisms.

[2] Pavlik I., Ulmann V., Falkinham J.O. (2022). Nontuberculous Mycobacteria: Ecology and Impact on Animal and Human Health. Microorganisms.

[3] Maleki M.R., Moaddab S.R. (2025). The growing impact of nontuberculous mycobacteria: A multidisciplinary review of ecology, pathogenesis, diagnosis, and treatment. Infect. Med..

[4] Daley C.L., Iaccarino J.M., Lange C., Cambau E., Wallace R.J., Andrejak C., Böttger E.C., Brozek J., Griffith D.E., Guglielmetti L. (2020). Treatment of Nontuberculous Mycobacterial Pulmonary Disease: An Official ATS/ERS/ESCMID/IDSA Clinical Practice Guideline. Clin. Infect. Dis..

[5] Kaczmarkowska A., Didkowska A., Kwiecień E., Stefańska I., Rzewuska M., Anusz K. (2022). The *Mycobacterium avium* complex—An underestimated threat to humans and animals. Ann. Agric. Environ. Med..

[6] Pedersen A.A., Løkke A., Fløe A., Ibsen R., Johansen I.S., Hilberg O. (2024). Nationwide Increasing Incidence of Nontuberculous Mycobacterial Diseases Among Adults in Denmark: Eighteen Years of Follow-Up. Chest.

[7] Hamaguchi Y., Morimoto K., Mitarai S. (2025). Laboratory-based surveillance of nontuberculous mycobacterial pulmonary disease in Japan. ERJ Open. Res..

[8] Zhang H., Tang M., Li D., Xu M., Ao Y., Lin L. (2024). Applications and advances in molecular diagnostics: Revolutionizing non-tuberculous mycobacteria species and subspecies identification. Front. Public Health.

[9] Cristancho-Rojas C., Varley C.D., Lara S.C., Kherabi Y., Henkle E., Winthrop K.L. (2024). Epidemiology of *Mycobacterium abscessus*. Clin. Microbiol. Infect..

[10] Johnston J.C., Chiang L., Elwood K. (2017). *Mycobacterium* *kansasii*. Microbiol. Spectr..

[11] Zulu M., Monde N., Nkhoma P., Malama S., Munyeme M. (2021). Nontuberculous Mycobacteria in Humans, Animals, and Water in Zambia: A Systematic Review. Front. Trop. Dis..

[12] Conyers L.E., Saunders B.M. (2024). Treatment for non-tuberculous mycobacteria: Challenges and prospects. Front. Microbiol..

[13] Tursun E.G., Bozok T., Aslan G. (2025). Antimicrobial resistance mechanisms in non-tuberculous mycobacteria. Folia Microbiol..

[14] Baethge C., Goldbeck-Wood S., Mertens S. (2019). SANRA-a scale for the quality assessment of narrative review articles. Res. Integr. Peer Rev..

[15] Ferrari R. (2015). Writing narrative style literature reviews. Med. Writ..

[16] Dowdell K., Haig S.J., Caverly L.J., Shen Y., LiPuma J.J., Raskin L. (2019). Nontuberculous mycobacteria in drinking water systems—The challenges of characterization and risk mitigation. Curr. Opin. Biotechnol..

[17] Loret J.F., Dumoutier N. (2019). Non-tuberculous mycobacteria in drinking water systems: A review of prevalence data and control means. Int. J. Hyg. Environ. Health.

[18] Haller S., Höller C., Jacobshagen A., Hamouda O., Abu Sin M., Monnet D.L., Plachouras D., Eckmanns T. (2016). Contamination during production of heater-cooler units by *Mycobacterium chimaera* potential cause for invasive cardiovascular infections: Results of an outbreak investigation in Germany, April 2015 to February 2016. Eurosurveillance.

[19] Perkins K.M., Lawsin A., Hasan N.A., Strong M., Halpin A.L., Rodger R.R., Moulton-Meissner H., Crist M.B., Schwartz S., Marders J. (2016). Notes from the Field: *Mycobacterium chimaera* Contamination of Heater-Cooler Devices Used in Cardiac Surgery—United States. MMWR Morb. Mortal. Wkly. Rep..

[20] Sommerstein R., Hasse B., Marschall J., Sax H., Genoni M., Schlegel M., Widmer A.F., the Swiss Chimaera Taskforce (2018). Global Health Estimate of Invasive *Mycobacterium chimaera* Infections Associated with Heater-Cooler Devices in Cardiac Surgery. Emerg. Infect. Dis..

[21] Sommerstein R., Schreiber P.W., Diekema D.J., Edmond M.B., Hasse B., Marschall J., Sax H. (2017). *Mycobacterium chimaera* Outbreak Associated with Heater-Cooler Devices: Piecing the Puzzle Together. Infect. Control Hosp. Epidemiol..

[22] Dávalos A.F., Garcia P.K., Montoya-Pachongo C., Rengifo A., Guerrero D., Díaz-Ordoñez L., Díaz G., Ferro B.E. (2021). Identification of Nontuberculous Mycobacteria in Drinking Water in Cali, Colombia. Int. J. Environ. Res. Public Health.

[23] Cirillo J.D., Falkow S., Tompkins L.S., Bermudez L.E. (1997). Interaction of *Mycobacterium avium* with environmental amoebae enhances virulence. Infect. Immun..

[24] Ovrutsky A.R., Chan E.D., Kartalija M., Bai X., Jackson M., Gibbs S., Falkinham J.O., Iseman M.D., Reynolds P.R., McDonnell G. (2013). Cooccurrence of free-living amoebae and nontuberculous Mycobacteria in hospital water networks, and preferential growth of *Mycobacterium avium* in *Acanthamoeba lenticulata*. Appl. Environ. Microbiol..

[25] Abukhalid N., Islam S., Ndzeidze R., Bermudez L.E. (2021). *Mycobacterium avium* Subsp. *hominissuis* Interactions with Macrophage Killing Mechanisms. Pathogens.

[26] Greub G., Raoult D. (2004). Microorganisms resistant to free-living amoebae. Clin. Microbiol. Rev..

[27] Thomson R., Tolson C., Carter R., Coulter C., Huygens F., Hargreaves M. (2013). Isolation of nontuberculous mycobacteria (NTM) from household water and shower aerosols in patients with pulmonary disease caused by NTM. J. Clin. Microbiol..

[28] Shen Y., Haig S.J., Prussin A.J., LiPuma J.J., Marr L.C., Raskin L. (2022). Shower water contributes viable nontuberculous mycobacteria to indoor air. PNAS Nexus.

[29] Barandiaran S., Ponce L., Piras I., Rosas A.C., Peña Martinez J., Marfil M.J. (2024). Detection of non-tuberculous mycobacteria in native wildlife species at conservation risk of Argentina. Front. Vet. Sci..

[30] Varela-Castro L., Barral M., Arnal M.C., Fernández de Luco D., Gortázar C., Garrido J.M., Sevilla I.A. (2022). Beyond tuberculosis: Diversity and implications of non-tuberculous mycobacteria at the wildlife-livestock interface. Transbound. Emerg. Dis..

[31] Gomez-Buendia A., Ortega J., Diez-Guerrier A., Rendahl A., Saez J.L., Bezos J., Romero B., Alvarez J. (2024). Evaluating the ability of non-tuberculous mycobacteria to induce non-specific reactions in bovine tuberculosis diagnostic tests in guinea pigs and cattle. Vet. Microbiol..

[32] Reil I., Špičić S., Barbić L., Duvnjak S., Kompes G., Benić M., Stojević D., Cvetnić Ž., Arapović J., Zdelar-Tuk M. (2023). Antimicrobial Resistance in Rapidly Growing Nontuberculous Mycobacteria among Domestic and Wild Animals Emphasizing the Zoonotic Potential. Microorganisms.

[33] Reil I., Barbić L., Kompes G., Zdelar Tuk M., Duvnjak S., Cvetnić Ž., Habrun B., Arapović J., Špičić S. (2023). Risk of zoonoses involving slow-growing non-tuberculous mycobacteria: Survey of antimicrobial resistance among strains from domestic and wild animals. J. Glob. Antimicrob. Resist..

[34] Reil I., Duvnjak S., Špičić S., Kompes G., Bagarić A., Đuras M., Gudan Kurilj A., Lukač M., Jelić M., Zdelar-Tuk M. (2024). Isolation of Multidrug-Resistant *Mycobacterium Avium* Subsp. *Avium* from a Wild Eurasian Otter (*Lutra lutra*). Antibiotics.

[35] Reil I., Špičić S., Kompes G., Duvnjak S., Zdelar-Tuk M., Stojević D., Cvetnić Ž. (2017). Nontuberculous mycobacteria in captive and pet reptiles. Acta Vet. Brno.

[36] Delghandi M.R., El-Matbouli M., Menanteau-Ledouble S. (2020). Mycobacteriosis and Infections with Non-tuberculous Mycobacteria in Aquatic Organisms: A Review. Microorganisms.

[37] Maboni G., Prakash N., Moreira M.A.S. (2024). Review of methods for detection and characterization of non-tuberculous mycobacteria in aquatic organisms. J. Vet. Diagn. Investig..

[38] Marianelli C., Pavlik I., Ghielmetti G. (2024). Editorial: Nontuberculous mycobacterial infections in animals and humans: Pathogenesis, diagnosis, prevention, treatment, and epidemiology. Front. Vet. Sci..

[39] Canetti D., Riccardi N., Antonello R.M., Nozza S., Sotgiu G. (2022). *Mycobacterium marinum*: A brief update for clinical purposes. Eur. J. Intern. Med..

[40] Sarsour R., Guirgus M., Rice R.C., Cappiello M., Damodaran C. (2025). *Mycobacterium marinum* Infection and Aquatic Exposure: A Clinical Reasoning Pathway. Cureus.

[41] Wei Y., Zhang J., Xin X., Wu Y., Wang L., Guo K., Xu Y., He S. (2024). Identification of *Mycobacterium marinum* in subcutaneous abscesses of an infected patient’s foot. J. Infect. Dev. Ctries..

[42] Meliefste H.M., Mudde S.E., Ammerman N.C., de Steenwinkel J.E.M., Bax H.I. (2024). A laboratory perspective on *Mycobacterium abscessus* biofilm culture, characterization and drug activity testing. Front. Microbiol..

[43] Oschmann-Kadenbach A.M., Schaudinn C., Borst L., Schwarz C., Konrat K., Arvand M., Lewin A. (2024). Impact of Mycobacteroides abscessus colony morphology on biofilm formation and antimicrobial resistance. Int. J. Med. Microbiol..

[44] Narimisa N., Bostanghadiri N., Goodarzi F., Razavi S., Jazi F.M. (2024). Prevalence of *Mycobacterium kansasii* in clinical and environmental isolates, a systematic review and meta-analysis. Front. Microbiol..

[45] Tobias Cudahy P.G., Liu P.C., Warren J.L., Sobkowiak B., Yang C., Ioerger T.R., Wu C.Y., Lu P.L., Wang J.Y., Chang H.H. (2024). Phylogeographic Analysis of *Mycobacterium kansasii* Isolates from Patients with *M. kansasii* Lung Disease in Industrialized City, Taiwan. Emerg. Infect. Dis..

[46] Morgado S., Ramos N.V., Freitas F., da Fonseca É.L., Vicente A.C. (2022). *Mycolicibacterium fortuitum* genomic epidemiology, resistome and virulome. Mem. Inst. Oswaldo Cruz.

[47] Wang J., Huang J., Peng S., Li L., Zhong K., Chen T. (2022). A clinical case and a review of *Mycobacterium fortuitum* infections direct diagnosis approach and treatment in a patient with leg fractures. J. Infect. Dev. Ctries..

[48] Mugetti D., Tomasoni M., Pastorino P., Esposito G., Menconi V., Dondo A., Prearo M. (2022). Reply to Pavlik et al. Clinical Relevance and Environmental Prevalence of *Mycobacterium fortuitum* Group Members. Comment on “Mugetti et al. Gene Sequencing and Phylogenetic Analysis: Powerful Tools for an Improved Diagnosis of Fish Mycobacteriosis Caused by *Mycobacterium fortuitum* Group Members. *Microorganisms* 2021, *9*, 797”. Microorganisms.

[49] Vahid S., Yan M., Turvey S.L. (2025). Review of the Canadian Nontuberculous Mycobacterial Disease Landscape-Challenges and Opportunities. Trop. Med. Infect. Dis..

[50] Kumar K., Ponnuswamy A., Capstick T.G., Chen C., McCabe D., Hurst R., Morrison L., Moore F., Gallardo M., Keane J. (2024). Non-tuberculous mycobacterial pulmonary disease (NTM-PD): Epidemiology, diagnosis and multidisciplinary management. Clin. Med..

[51] Kravvas G., Aboukhatwah N., Meghoma L., Vilenchik V., Oxley J., Keith D. (2024). A Novel, Nonaquatic Zoonotic Transmission of *Mycobacterium marinum*. Case Rep. Infect. Dis..

[52] Shi X., Zhang Y., Jin N., Guo W., Hao K., Ren X., Yeet S. (2025). Mycobacteriosis caused by *Mycobacterium marinum* in *Pterophyllum scalare*: First evidence from China. Anim. Dis..

[53] Gross J.E., Finklea J.D., Caceres S.M., Poch K.R., Hasan N.A., Jia F., Epperson L.E., Lipner E.M., Vang C.K., Honda J.R. (2024). Genomic epidemiology of *Mycobacterium abscessus* at an adult cystic fibrosis programme reveals low potential for healthcare-associated transmission. ERJ Open Res..

[54] Aitken M.L., Limaye A., Pottinger P., Whimbey E., Goss C.H., Tonelli M.R., Cangelosi G.A., Dirac M.A., Olivier K.N., Brown-Elliott B.A. (2012). Respiratory outbreak of *Mycobacterium abscessus* subspecies massiliense in a lung transplant and cystic fibrosis center. Am. J. Respir. Crit. Care Med..

[55] Bryant J.M., Grogono D.M., Greaves D., Foweraker J., Roddick I., Inns T., Reacher M., Haworth C.S., Curran M.D., Harris S.R. (2013). Whole-genome sequencing to identify transmission of *Mycobacterium abscessus* between patients with cystic fibrosis: A retrospective cohort study. Lancet.

[56] Nguyen M.H., Haas M.K., Kasperbauer S.H., Calado Nogueira de Moura V., Eddy J.J., Mitchell J.D., Khare R., Griffith D.E., Chan E.D., Daley C.L. (2024). Executive Summary: State-of-the-Art Review: Nontuberculous Mycobacterial Pulmonary Disease: Patients, Principles, and Prospects. Clin. Infect. Dis..

[57] Nguyen T.Q., Heo B.E., Jeon S., Ash A., Lee H., Moon C., Jang J. (2024). Exploring antibiotic resistance mechanisms in *Mycobacterium abscessus* for enhanced therapeutic approaches. Front. Microbiol..

[58] Kurz S.G., Zha B.S., Herman D.D., Holt M.R., Daley C.L., Ruminjo J.K., Thomson C.C. (2020). Summary for Clinicians: 2020 Clinical Practice Guideline Summary for the Treatment of Nontuberculous Mycobacterial Pulmonary Disease. Ann. Am. Thorac. Soc..

[59] Clinical and Laboratory Standards Institute (2018). Susceptibility Testing of Mycobacteria, Nocardia spp., and Other Aerobic Actinomycetes (M24).

[60] Clinical and Laboratory Standards Institute (2018). Performance Standards for Susceptibility Testing of Mycobacteria, Nocardia spp., and Other Aerobic Actinomycetes (M62).

[61] EUCAST (AMST) (2014). Mycobacteria (Antimycobacterial Susceptibility Testing Subcommittee).

[62] Fröberg G., Maurer F.P., Chryssanthou E., Fernström L., Benmansour H., Boarbi S., Mengshoel A.T., Keller P.M., Viveiros M., Machado D. (2023). EUCAST AMST and ESCMYC study groups. Towards clinical breakpoints for non-tuberculous mycobacteria—Determination of epidemiological cut off values for the *Mycobacterium avium* complex and *Mycobacterium abscessus* using broth microdilution. Clin. Microbiol. Infect..

[63] Solanki P., Lipman M., McHugh T.D., Satta G. (2022). Whole genome sequencing and prediction of antimicrobial susceptibilities in non-tuberculous mycobacteria. Front. Microbiol..

[64] Hou Y., Pi R., Jia J., Wu Z., Huo F., Zhou Y., Jiang H., Takiff H.E., Zhu C., Wang W. (2025). Limited predictive power of known resistance genes for phenotypic drug resistance in clinical *Mycobacterium abscessus* complex from Beijing in China. Antimicrob. Agents Chemother..

[65] APHL (2025). Nontuberculous Mycobacteria: An Emerging Public Health Concern.

[66] CDC (2026). Nontuberculous Mycobacteria Surveillance Program.

[67] Prevots D.R., Loddenkemper R., Sotgiu G., Migliori G.B. (2017). Nontuberculous mycobacterial pulmonary disease: An increasing burden with substantial costs. Eur. Respir. J..

[68] Winthrop K.L., Henkle E., Walker A., Cassidy M., Hedberg K., Schafer S. (2017). On the Reportability of Nontuberculous Mycobacterial Disease to Public Health Authorities. Ann. Am. Thorac. Soc..

[69] Sharma S.K., Upadhyay V., Mohan A. (2026). From anonymity to stardom: History of nontuberculous mycobacterial disease in humans. Front. Cell. Infect. Microbiol..

[70] Lemson A., van Laarhoven A., Kurver L., Stemkens R., Aarnoutse R., Boeree M., van Ingen J., Hoefsloot W. (2025). Treatment of nontuberculous mycobacterial pulmonary disease requires a stepwise and multidisciplinary approach. Expert. Rev. Respir. Med..

[71] Saminathan G., Kumar V., Shanmugam S., Rajendran P. (2025). Revolutionizing non-tuberculous mycobacteria research: Advances in molecular diagnostics, phylogenetics, and clinical management. J. Microbiol. Methods..

[72] Mohapatra P.R., Mishra B. (2025). Nontuberculous mycobacteria: An update. Indian J. Tuberc..

[73] Dohál M., Wetzstein N., Hromádková M., Mäsiarová S., Rasmussen E.M., Kunč P., Škereňová M., Porvazník I., Solovič I., Niemann S. (2025). Diagnostics, resistance and clinical relevance of non-tuberculous mycobacteria unidentified at the species level by line probe assays: A bi-national study. Ann. Clin. Microbiol. Antimicrob..

[74] Verma D., Chan E.D., Ordway D.J. (2020). Non-Tuberculous Mycobacteria Interference with BCG-Current Controversies and Future Directions. Vaccines.

[75] Ghasemi F., Kardan-Yamchi J., Heidary M., Karami-Zarandi M., Akrami S., Maleki A., Khoshnood S., Kazemian H. (2024). Effects of non-tuberculous mycobacteria on BCG vaccine efficacy: A narrative review. J. Clin. Tuberc. Other Mycobact. Dis..

[76] Poyntz H.C., Stylianou E., Griffiths K.L., Marsay L., Checkley A.M., McShane H. (2014). Non-tuberculous mycobacteria have diverse effects on BCG efficacy against *Mycobacterium tuberculosis*. Tuberculosis.

