# Supplementary material for: Non-Tuberculous Mycobacteria at the Human–Animal–Environment Interface: Antimicrobial Resistance, Environmental Persistence and Cross-Species Exposure Risks
Source: Antibiotics (Basel). 2026 May 5;15(5):467. doi: 10.3390/antibiotics15050467 (PMC13203507; doi:10.3390/antibiotics15050467)
Supplement: Supplementary file 1 [file antibiotics-15-00467-s001.zip › antibiotics-4286828-supplementary.pdf]

Supplementary Materials

Table S1. Representative database search strings used in this narrative review

Database	Representative search strategy	Final search date
**PubMed**	("nontuberculous mycobacteria"[Title/Abstract] OR "non-tuberculous mycobacteria"[Title/Abstract] OR NTM[Title/Abstract]) AND ("environmental reservoirs"[Title/Abstract] OR "drinking water"[Title/Abstract] OR "water systems"[Title/Abstract] OR biofilm*[Title/Abstract] OR soil[Title/Abstract] OR aerosol*[Title/Abstract] OR sediment*[Title/Abstract] OR animal*[Title/Abstract] OR livestock[Title/Abstract] OR wildlife[Title/Abstract] OR "companion animals"[Title/Abstract] OR reptile*[Title/Abstract] OR fish[Title/Abstract] OR aquaculture[Title/Abstract] OR "One Health"[Title/Abstract] OR "antimicrobial resistance"[Title/Abstract] OR AMR[Title/Abstract] OR "intrinsic resistance"[Title/Abstract] OR efflux[Title/Abstract] OR "macrolide resistance"[Title/Abstract] OR "erm(41)"[Title/Abstract] OR "whole-genome sequencing"[Title/Abstract] OR WGS[Title/Abstract] OR "genotype-phenotype"[Title/Abstract])	24 April 2026
**Scopus**	TITLE-ABS-KEY(("nontuberculous mycobacteria" OR "non-tuberculous mycobacteria" OR NTM) AND ("environmental reservoirs" OR "drinking water" OR "water systems" OR biofilm* OR soil OR aerosol* OR sediment* OR animal* OR livestock OR wildlife OR "companion animals" OR reptile* OR fish OR aquaculture OR "One Health" OR "antimicrobial resistance" OR AMR OR "intrinsic resistance" OR efflux OR "macrolide resistance" OR "erm(41)" OR "whole-genome sequencing" OR WGS OR "genotype-phenotype"))	24 April 2026
**Web of Science**	TS=("nontuberculous mycobacteria" OR "non-tuberculous mycobacteria" OR NTM) AND TS=("environmental reservoirs" OR "drinking water" OR "water systems" OR biofilm* OR soil OR aerosol* OR sediment* OR animal* OR livestock OR wildlife OR "companion animals" OR reptile* OR fish OR aquaculture OR "One Health" OR "antimicrobial resistance" OR AMR OR "intrinsic resistance" OR efflux OR "macrolide resistance" OR "erm(41)" OR "whole-genome sequencing" OR WGS OR "genotype-phenotype")	24 April 2026

Note: Additional targeted searches were performed for clinically and epidemiologically relevant taxa and settings, including MAC, *M. abscessus*, *M. kansasii*, *M. marinum*, bovine tuberculosis diagnostic interference, hospital water systems, and *M. chimaera* heater-cooler device outbreaks.
